# A new era is emerging at scientific user facilities

**DOI:** 10.1107/S2052252523010928

**Published:** 2024-01-01

**Authors:** Dimitri Argyriou

**Affiliations:** a Advanced Light Source, Lawrence Berkeley National Laboratory, Berkeley, CA 94720-8229, USA

**Keywords:** scientific user facilities, COVID-19 pandemic, telepresence, artificial intelligence (AI), hybrid working, editorial

## Abstract

Global scientific exchange has been profoundly perturbed by the COVID-19 pandemic, altering user travel behaviours and accelerating the use of remote access. Combined with the advent of artificial intelligence (AI), these trends together can change how large-scale user scientific facilities are used by the community and managed by operators.

One of the features of the culture of science is the exchange of ideas and the sharing of advances. This typically happens by scientists and their students travelling to conferences, collaborating labs or large-scale user facilities to participate and contribute to discussions and experiments. Such travel is a wonderful way to not only expose each other to new work and ideas but also shape and share culture and habits. Most importantly it has been a great way to educate and train the next generation of scientists to the scientific method and to advance ideas, techniques and equipment.

However, this global scientific exchange has been profoundly perturbed by the COVID-19 pandemic. Changes in user travel behaviours, the use of remote access greatly perfected and adopted during the pandemic, combined with the advent of remarkable advances in artificial intelligence (AI), are aligning in such a way that they may possibly change how large-scale scientific facilities operate. This transformation is still ongoing and the new normal is still evolving.

Most scattering facilities report that while the number of submitted user proposals for experiments is slowly recovering toward pre-pandemic levels, the physical presence of users is recovering at a much slower pace while telepresence is increasing. The implications of this pattern are significant for facility operators. Experiments that were once performed by the users themselves are now dependent more so than before on the local beamline staff, thus pressuring the facility’s operational staff levels. And, while one can argue that remote presence in experiments may indeed expand the user base of facilities, the reality is that this emerging user base may be less able to conduct experiments in the future as students are not hands-on trained as rapidly as in the past. This again places increasing pressure on the local staff needed to support physically experiments at the facility.

This shift in user behaviour is taking place while scientific facilities are generating increasing amounts of data and experiments are becoming more demanding. Upgrades to ESRF and LCLS and planned upgrades at APS, ALS and Diamond facilities as well as the many new facilities that are coming online such as ESS, will add even further to the significant deluge of high-quality data. Innovations in the manipulation of the radiation, use of complex sample environments and other add-ons at the beamlines increase the demands on the experimental team to provide real-time support during the experiment as well as substantial support in the processing and analysis of the data post-experiment.

So how do facilities adjust to the reality that while experiments are growing more demanding and generating more data, user physical participation is slow to recover and shifting more to telepresence?

There is no doubt in my mind that the evolving trend at photon and neutron facilities is destined towards a hybrid model — a blend of traditional onsite work with enhanced remote participation in experiments. Already many facilities are investing heavily to enable this mode of user engagement. While the pandemic accelerated the adoption of remote access and AI tools, the value of in-person collaboration and hands-on experimentation remains irreplaceable. The future most likely holds a coexistence, leveraging the strengths of both virtual and physical realms to drive scientific progress. AI will also be part of that future and this will further add to the shift in the mix of skills needed at the beamline to incorporate the AI/data expertise needed when running an experiment, alongside the increasing numbers of beamline scientists who will be needed to mitigate the smaller number of physically participating users.

The future, where a user proposal will be read by an AI chatbot, its content and purpose understood sufficiently to configure a beamline and predict the initial data from the sample examined, is well within reach (Hexemer *et al.*, 2024 ▸). An autonomous AI-driven beamline that will perform routine measurements for users is not that far away either.

So where does that leave user facilities, that community watering hole, bringing scientists and ideas together, fostering collaboration and scientific progress?

Here, we need to remember that the science is in the sample. Scientists who made and understand the sample are irreplaceable when conducting experiments and their collaboration with the beamline staff is essential to success. It is no surprise therefore that the scientific community remains deeply committed and engaged in the future of large-scale facilities. A recent personal example was the user meeting of the Advanced Light Source which focused on the capabilities that users will harness with its upcoming upgrade. It is clear that the user community fully recognizes the value brought by user facilities and is fully invested in their success, irrespective of changing trends in user presence.

The brightest future lies in fully embracing AI to harness automation and autonomy at beamlines where it is useful and adds operational value. Deploying AI in harvesting all essence from the PTBs of data collected is unavoidable, given the volumes that we’ll soon face. Remote participation in experiments must be supplemented by hands-on training to provide sufficient experience to users so that the results obtained are put into the right context. Nothing still replaces the value of getting your *hands dirty* on a beamline and engaging in strong collaborations with the local staff.

The COVID-19 pandemic together with the advent of AI is acting as a catalyst for change within large-scale scientific facilities, reshaping user behaviours and accelerating the role of AI in beamline automation and the analysis of ever-growing volume of data. As we navigate this transformative landscape, we cannot take much of the past for granted. Training of the next generation of users needs to be a community priority. We need to find new ways to support collaboration and user engagement at the beamline so as to perform the most challenging and impactful experiments and drive innovation. Incorporating AI-driven advances to deal with data will be needed to harvest value from experiments and fuel future breakthroughs. To succeed, facilities will need to collaborate widely with the AI community, access infrastructures to manage and process data and work with sponsors to manage the shift in skills needed in staff at the beamline. This is a significant transformation that is best embraced head-on.
